# Comparative Analysis of Atherogenic Lipoproteins L5 and Lp(a) in Atherosclerotic Cardiovascular Disease

**DOI:** 10.1007/s11883-024-01209-3

**Published:** 2024-05-16

**Authors:** Omer Akyol, Chao-Yuh Yang, Darren G. Woodside, Huan-Hsing Chiang, Chu-Huang Chen, Antonio M. Gotto

**Affiliations:** 1 Molecular Cardiology Research Laboratories, Vascular and Medicinal Research, The Texas Heart Institute, Houston, Texas 77030 USA; 2 Department of Medicine, Baylor College of Medicine, One Baylor Plaza, Houston, Texas 77030 USA; 3 Molecular Cardiology Research Laboratories, The Texas Heart Institute, Houston, TX 77030 USA; 4 Weill Cornell Medical College, New York, NY 10021 USA

**Keywords:** Lp(a), L5, Electronegative LDL, Atherogenesis, Atherosclerotic Cardiovascular Disease, Oxidized LDL

## Abstract

**Purpose of Review:**

Low-density lipoprotein (LDL) poses a risk for atherosclerotic cardiovascular disease (ASCVD). As LDL comprises various subtypes differing in charge, density, and size, understanding their specific impact on ASCVD is crucial. Two highly atherogenic LDL subtypes—electronegative LDL (L5) and Lp(a)—induce vascular cell apoptosis and atherosclerotic changes independent of plasma cholesterol levels, and their mechanisms warrant further investigation. Here, we have compared the roles of L5 and Lp(a) in the development of ASCVD.

**Recent Findings:**

Lp(a) tends to accumulate in artery walls, promoting plaque formation and potentially triggering atherosclerosis progression through prothrombotic or antifibrinolytic effects. High Lp(a) levels correlate with calcific aortic stenosis and atherothrombosis risk. L5 can induce endothelial cell apoptosis and increase vascular permeability, inflammation, and atherogenesis, playing a key role in initiating atherosclerosis. Elevated L5 levels in certain high-risk populations may serve as a distinctive predictor of ASCVD.

**Summary:**

L5 and Lp(a) are both atherogenic lipoproteins contributing to ASCVD through distinct mechanisms. Lp(a) has garnered attention, but equal consideration should be given to L5.

## Introduction

Despite aggressive lipid-lowering strategies targeting plasma low-density lipoprotein cholesterol (LDL-C) concentrations, atherosclerotic cardiovascular disease (ASCVD) persists [1]. This therapeutic inadequacy underscores the ongoing quest to identify key atherogenic LDL entities in lipid research and clinical medicine.

Among these atherogenic entities, small-dense LDL (sdLDL) and lipoprotein (a) [Lp(a)] have garnered significant attention. Clinical observations link sdLDL to increased ASCVD risks, although mechanistic evidence remains limited [2••]. Similarly, Lp(a) is positively correlated with ASCVD, albeit with a more restricted prognostic value due to its predominantly genetically determined plasma levels [3]. Another atherogenic LDL variant is oxidized LDL (oxLDL), formed through LDL oxidation; however, direct analysis of circulating oxLDL is challenging.

Through anion-exchange chromatography, LDL can be categorized into five subclasses (L1–L5) with L5 having the highest electronegativity [4]. L5 remains unoxidized *in vivo* yet demonstrates toxicity toward endothelial cells (ECs), other vascular cells, and cardiomyocytes, at levels similar to those of oxLDL. The oxidized status is a main differentiating factor between oxLDL and L5.

Lp(a) is a form of LDL bound to apolipoprotein (a) [apo(a)]. Apo(a) shares structural similarity with plasminogen, thus indirectly interfering with fibrinolysis by inhibiting the conversion of plasminogen to plasmin [5]. Chemical analysis has revealed that L5 is associated with apo(a), which may contribute to L5’s thrombogenic effects [6]. Unlike Lp(a), L5 particles are more heterogeneous, including associations with apoE, apoCIII, apoA1; L5’s modifications include glycosylation and elevated levels of triacylglycerols (TG) and cholesterol esters, all of which enhance its electronegativity and atherogenicity [6].

Although oxLDL, sdLDL, Lp(a), or L5 has not emerged as the singular "culprit" responsible for the atherogenic and thrombogenic processes associated with LDL, Lp(a) and L5 share chemical and functional similarities and are most likely the entities to act either alone or synergistically to cause cardiovascular damage. Both modified forms of LDL warrant equal attention in identifying targets for effective treatment regimens.

## Apolipoprotein Content of L5 and Lp(a)

**Fig. 1:**
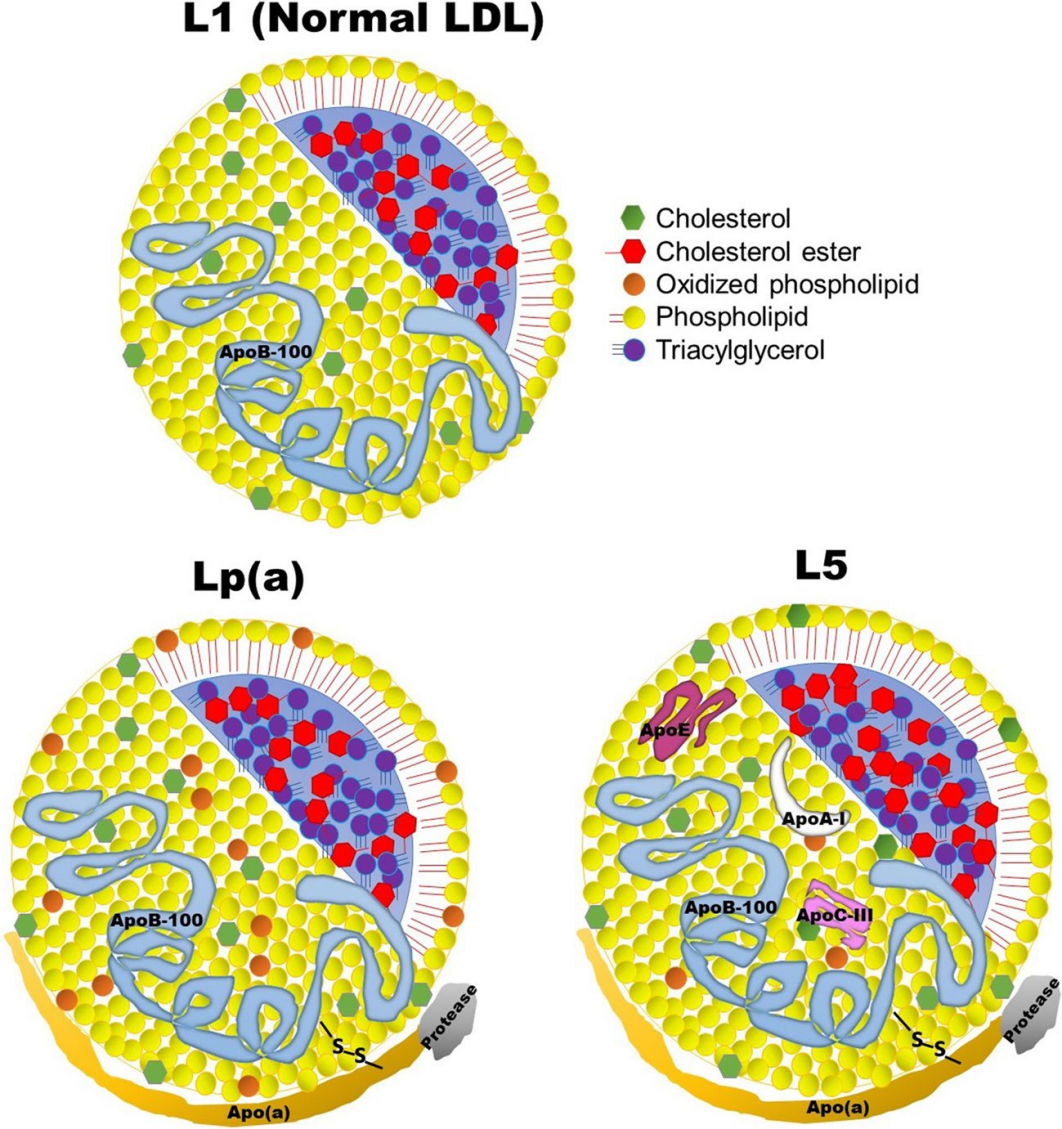
Schematic representation of the structural components of L1, L5, and Lp(a). Normal LDL is represented by L1 because they have the same chemical structure and function. Apo(a) is noted to be richly associated with oxidized phospholipids, whereas L5 is as minimally oxidized as L1, if any. Unlike L1, Lp(a) and L5 have two disulfide-linked apolipoproteins: apoB100 and apo(a). L5 has a lipid core composed of greater amounts of cholesteryl ester than triacylglycerols. Apo, apolipoprotein; LDL, low-density lipoprotein; Lp(a), lipoprotein(a)

First detected by Berg in 1963 [7], Lp(a) distinguishes itself from normal LDL by containing an apo(a) molecule covalently attached to apoB100 via a disulfide bond (Fig. 1). Synthesized in the liver, apo(a) is a glycoprotein with 6 to 14 genetic isoforms containing highly glycosylated, disulfide-stabilized kringle domains [5].

Lp(a)’s structural component apo(a) has amino acid sequence homology to plasminogen; however, an arginine-to-serine substitution bars its conversion into an active enzyme. This prevents Lp(a) from playing a direct role in the degradation of fibrin. However, as it can bind plasminogen activators and fibrin, it can impede fibrinolysis by preventing the formation of plasmin and competing with plasminogen binding to fibrin [5]. Although its other potential atherogenic mechanisms are less well developed, Lp(a) has clearly been implicated in the pathophysiology of ASCVD.

L5 is a highly electronegative form of LDL (Fig. 1) that is abundant in patients with hypercholesterolemia and various cardiovascular and metabolic diseases, but not in healthy individuals. Like Lp(a), L5 particles contain apoB100, along with several other apolipoproteins including apolipoprotein E (apoE), apoAI, apoCIII, and apo(a) (Fig. 1). Apolipoprotein heterogeneity increases from L1 to L5. In the least electronegative subfraction of LDL (L1), the protein framework almost entirely comprises apoB100 (isoelectric point (pI): 6.6). L5 contains additional apolipoproteins with lower isoelectric points, including apo(a) (pI: 5.5), apoAI (pI: 5.4), apoE (pI: 5.5), and apoCIII (pI: 5.1) [6]. Because proteins are negatively charged at a pH above their pI, the relatively low average pI of the apoprotein constituents of L5 helps maintain this particle in a negatively charged state at physiological pH. Liquid chromatography studies have shown that the protein framework of L1 contains approximately 99.7% apoB100 (of total protein weight), with small quantities of apoE, albumin, and apoAI [6]. In contrast, L5 consists of approximately 61% apoB100, about 30% apo(a), 2% apoAI, and 3% apoE. Additionally, L5 contains apoCIII, albumin, apoJ, paraoxonase1, and platelet activating factor-acetyl hydrolase (PAF-AH).

## Chemical and Biochemical Characteristics of Lp(a) and L5

Lp(a) particles are spherical macromolecular complexes approximately 24-28.3 nm in diameter that have a density between 1.064 - 1.101 g/mL (Table 1) [8]. Apo(a) polymorphisms contribute to Lp(a) size heterogenicity as apo(a) can have from 15 to 37 kringle IV-like repeats [9]. Lp(a) is one of the main carriers of oxidized phospholipids (oxPL) in human plasma; oxPL mediate numerous cellular and molecular events underlying atherothrombotic cardiovascular disease (CVD) and calcific aortic valve injury [10].

**Table 1:** Characteristics of human plasma L5, Lp(a), and regular LDL (L1)

	Density (g/mL)	Molecular weight (Da)	Diameter (nm)	Electrophoretic mobility	Major proteins	Major lipids
Lp(a)	1.064-1.101	2.9-3.7 x 10^6^	24-28.3	Pre-β	apo(a) apoB100	Cholesteryl esters Phospholipids
L5	1.019-1.063*	Not known	24-26.5	β	apo(a) apoAI apoB100 apoCIII apoE	Cholesterol Triacylglycerols
L1	1.019-1.063	2.75 x 10^6^	19-23	β	apoB100	Cholesteryl esters

*No data are found regarding the exact density of L5 particles. The values shown are an estimation.

Apo(a) is typically solvent exposed on Lp(a) particles and is linked to apoB100 via sulfhydryl group binding between Cys4326 of apoB100 and Cys4057 within the kringle IV9 of apo(a). In addition, apo(a) kringle domains noncovalently interact with apoB100 through lysine moieties (Lys680 and Lys4372) [11].

Susceptible to alterations induced by environmental factors, Lp(a) has the capacity for modifications in both particle diameter and noncovalent interactions between apo(a) and apoB100, thereby potentially augmenting its atherogenic propensity [12]. Artificially prepared oxidized Lp(a) [oxLp(a)] exhibits less effective lipid extraction by glycerol, indicating lipids are more tightly bound within particles. Oxidative changes could lead to the impaired recognition of oxLp(a) by low-density lipoprotein receptor (LDLR) and hinder the recognition and clearance of particles in the liver. If these changes occur *in vivo*, they may lead to lipid accumulation in vessel walls, foam cell production, and initiation and progression of atherosclerotic disease [13].

L5 has many physicochemical properties that diverge from those of traditionally described LDL. In terms of lipid composition, L5 has higher levels of TG, non-esterified fatty acids (NEFA), ceramide, and lysophosphatidylcholine (lysoPC) than does native LDL or L1. Additionally, L5 has phospholipolytic activity not seen in LDL. Because of its unique conformation of the amino-terminal region of apoB100, L5 has a strong association with mucoproteins in the extracellular matrix and on cell surfaces. This property and its extensive accumulation of proteoglycans facilitate subendothelium retention of L5, potentially aiding in initiating fatty streak formation [14].

Proteomic studies have revealed that electronegative LDLs isolated from patients with familial hypercholesterolemia (FH) or diabetes mellitus (DM) contain lipoprotein-related phospholipase A2 (Lp-PLA2) [15, 16], which induces endothelial inflammation and pathologic changes that lead to ASCVD [17]. Quantitative analysis of electronegative LDL subfractions from patients with DM revealed that L5 contained one Lp-PLA2 moiety per 237 L5 particles, whereas L1 contained one Lp-PLA2 moiety per 152,000 L1 particles [15]. By cleaving phospholipids, Lp-PLA2 generates oxidized versions of proinflammatory lipid metabolites, namely NEFA and lysoPC [17]. The increased presence of Lp-PLA2 in L5 as compared to L1 may lead to elevated levels of NEFA and lysoPC and prevents L5 from effectively binding with PAF-AH, a potent immediate-response molecule. Notably, PAF-AH is 5-fold higher in L5 than in unsubfractionated LDL in human plasma [18]. Consequently, this contributes to the proinflammatory characteristics of electronegative LDL.

## Oxidized Products of Lp(a) and L5

Oxidized lipids, such as oxPL, are potent proinflammatory species formed by the oxidation of sn2-polyunsaturated fatty-acyl chains [19•]. When associated with Lp(a), they enhance Lp(a)’s ability to trigger proinflammatory responses in blood vessels [20••]. At high plasma concentrations, as in some genetically predisposed populations, Lp(a) transports these proinflammatory oxPL substances to the sites of injury, initiating the aggregation of Lp(a) particles and their association with various extracellular matrix elements [20••]. Around atherosclerotic lesions, inflammatory and oxidative stress reactions promote generation of reactive oxygen species (ROS), which facilitate cleavage of fatty acid acyl-chains to produce reactive moieties and promote the binding of newly formed oxPLs to Lp(a). The oxLp(a) formed is rapidly internalized by macrophages, leading to foam cell formation [21], initiating early atherogenic events [22].

L5 is a collection of electronegative LDL particles that undergo modification through multiple mechanisms. Of these L5 particles, oxLDL constitutes only a small and varying portion [23]. Since the components of L5 are not oxidatively altered, the increased electronegativity of L5 particles is attributed to other nonoxidative sources [24]. Charge heterogeneity in native LDL is not associated with lipid peroxidation or the derivatization of apoB100’s free amino groups. In contrast, LDL enriched with apolipoproteins other than apoB100 may in part explain particle charge variation, as in the case of L5 [25]. In brief, L5’s cytotoxicity is largely independent of its oxidative state.

## Metabolism of Lp(a) and L5

Levels of circulating Lp(a), primarily determined by genetic factors, remain stable throughout life, unlike other cholesterol-carrying apoB containing particles like LDL, which are affected by both genetics and lifestyle choices. The codominant expression of two *LPA* alleles determines plasma Lp(a) levels. However, certain features of the Lp(a) synthesis pathway are unknown. LDL is produced from VLDL, but Lp(a) is synthesized independently as a separate lipoprotein. Apo(a) is almost exclusively expressed in the liver [26]. Recent *in vivo* kinetic studies in humans have challenged the conventional notion that Lp(a) assembly is exclusively extracellular from newly synthesized apo(a) and circulating LDL. Instead, evidence suggests intracellular assembly through a complex process involving multiple non-covalent interactions between apo(a) and apoB100 of LDL [26, 27]. Plasma Lp(a) concentration is determined primarily by the number of apo(a) kringle domains. Because increased numbers of kringle domains impede Lp(a)’s release from the liver, most Lp(a) particles in the plasma are smaller isoforms [28].

Lp(a) particles have been proposed to bind to LDLR in the liver, albeit with low affinity [29]. Plasma Lp(a) is elevated in patients with homozygous or heterozygous FH with complete deletion of the LDLR gene [30]. Lp(a) is not reduced by statins but by PCSK9 inhibitors, which effectively inhibit LDLR degradation, further suggesting that the clearance of Lp(a) is LDLR-dependent. However, other lipid-lowering treatment modalities that have mechanisms of action not involving the LDLR pathway, such as cholesteryl ester transfer protein inhibitors and niacin inhibitors, also reduce plasma Lp(a). An alternative Lp(a) clearance pathway in the kidneys may account for 10% of total Lp(a) clearance [31]. In patients with chronic kidney disease, impaired renal clearance of Lp(a) rather than its formation results in increased Lp(a) levels [32].

How L5 is synthesized is unclear, but modifications other than LDL oxidation are involved. Exposure to heat (37°C) or copper mobilizes native LDL or L1 to the electronegative end of the chromatographic spectrum [33]; however, these *in vitro* observations likely do not extrapolate to *in vivo* mechanisms. Because glycosylation occurs on two residues of the associated apoE, the mechanism of L5’s synthesis partly involves enzymatic activities in hepatocytes [34]. The abundant TG content in L5 implies inefficient TG hydrolysis, a process regulated by extracellular lipoprotein lipase that may be compromised by apoCIII in L5 [35]. These observations suggest that L5 is a lipoprotein product regulated by both intra- and extracellular mechanisms.

L5 is not recognized by the LDLR but is instead internalized into ECs via the lectin-like oxidized LDL receptor (LOX-1), which is structurally associated with the family of C-type lectins that have a type-II transmembrane protein. LOX-1 also exhibits a strong affinity for various negatively charged particles or substances, including oxLDL, tumor necrosis factor-alpha (TNF-α), and C-reactive protein (CRP), due to its lectin component [36].

## Inflammatory Characteristics and Immune-related Actions of Lp(a) and L5

The pathogenicity of Lp(a) is related to immune cells and inflammation, as evidenced by its ability to drive the formation of inflammatory monocytes in bone marrow [37]. Not surprisingly, patients with increased Lp(a) levels have significantly higher levels of atypical CD14^+^CD16^++^ monocytes [38•]. The link between Lp(a) and the innate immune system stems from oxPL on Lp(a) being recognized by innate immune receptors [10]. The 2.5-fold increase in the neutrophil-to-lymphocyte ratio seen with increased plasma Lp(a) levels may increase the likelihood of major adverse cardiovascular events (MACE) in patients with early coronary heart disease symptoms by two-fold [39]. For example, patients with Lp(a) levels over 30 mg/dL, combined with either a high neutrophil-to-lymphocyte ratio or increased neutrophil count, experience a quick onset of MACE [39]. Some findings have supported that Lp(a) acts as an acute-phase reactant, as its concentration markedly increases after tissue injury [40]. Other studies have indicated an association between Lp(a) and inflammatory cytokines including TNF-α, transforming growth factor-β, IL-6, and monocyte chemoattractant protein-1 [41, 42]. The apo(a) gene contains numerous IL-6 response elements [43], and cell culture studies showed that IL-6 upregulates the expression of the apo(a) gene, leading to the accumulation of Lp(a) granules [44]. The inflammation-induced elevation of Lp(a) and the affinity of Lp(a) for extracellular matrix proteins may contribute to Lp(a) accumulation in arteries during early atherosclerosis.

L5 can activate various components of the innate immune response. L5 induces ECs to express vascular cell adhesion molecule-1 [45, 46], which recruits inflammatory cells into developing atherosclerotic plaques [47, 48]. Indeed, L5 derived from patients with FH induced mononuclear cell adhesion to ECs [23]. L5-activated vascular ECs release cytokines that act on other cells in the arterial wall. Chang et al. [49] demonstrated that L5 activates mitogen-activated protein kinases and nuclear factor kappa B (NF-κB) signaling pathways, leading to the production of IL-6, IL-1β, and TNF-α in macrophages derived from differentiated THP-1 cells. It is not clear if L5 induces increased IL-1β gene expression or activation of the NLRP3 inflammasome that generates mature IL-1β. Notably, targeting IL-1β with the monoclonal antibody canakinumab reduces cardiovascular events in patients with ASCVD [50], demonstrating a direct link between inflammation and atherosclerosis. Overall, these results suggest that electronegative LDLs promote atherogenesis by activating inflammatory pathways that contribute to the initiation and progression of ASCVD.

## Pathophysiologic Role of Lp(a) and L5 in Atherothrombotic Disease

**Fig 2:**
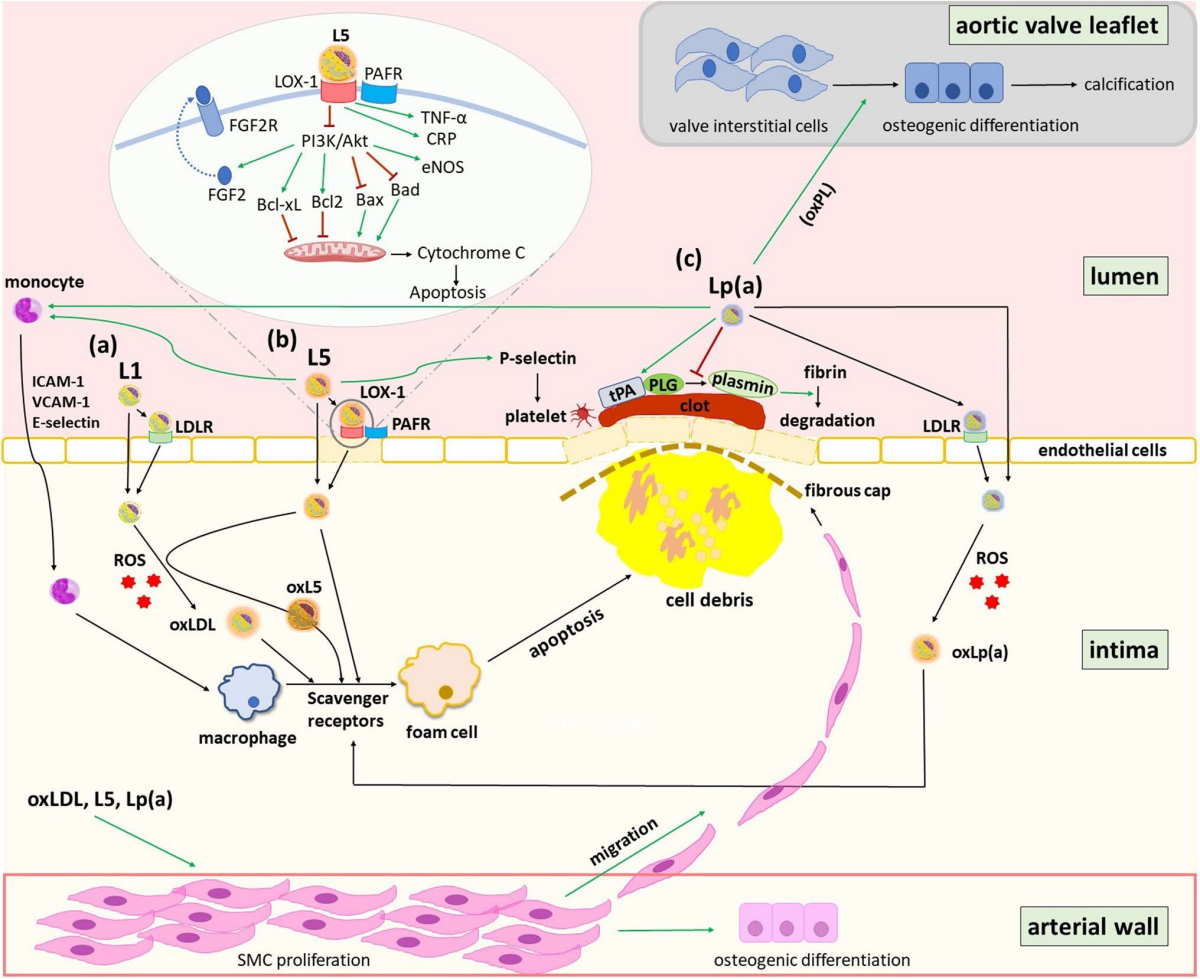
Schematic representation showing the contribution of L1, L5, and Lp(a) to atherosclerosis. (a) Under normal circumstances, LDLR mediates the uptake of LDL into ECs. The rate of production of oxLDL in the arterial intima *in vivo* is a function of the concentration of native LDL present. Macrophages are loaded with cholesterol as they take up all oxidized products including oxLDL, oxLp(a), and oxL5. Oxidized-LDL generated either locally or systemically stimulates ECs to express adhesion molecules, including ICAM-1, vascular cell adhesion molecule-1, and E-selectin, which are responsible for the adhesion of blood monocytes. (b) L5 activates ECs via LOX-1 and PAFR, suppressing PI3K/Akt signaling and increasing the release of TNF-α. L5 induces the expression of Bax and Bad, followed by the release of cytochrome c from mitochondria, thereby inducing apoptosis. L5 also augments adhesion between monocytes and ECs. (c) Lp(a) promotes EC damage by inducing mural thrombosis and EC dysfunction, leading to the passage of Lp(a) through the protective endothelial layer. Lp(a) can also activate circulating monocytes and induce monocyte trafficking to the arterial wall. The green arrows show induction/stimulation. Red bars show repression/prevention. Akt, protein kinase B; Bcl2, B cell lymphoma 2; CRP, C-reactive protein; ECs, endothelial cells; eNOS, endothelial nitric oxide synthase; FGF2, fibroblast growth factor 2; FGF2R, fibroblast growth factor 2 receptor; ICAM-1, intercellular adhesion molecule 1; LDL, low-density lipoprotein; LDLR, low-density lipoprotein receptor; LOX-1, lectin-like oxidized low-density lipoprotein receptor-1; Lp(a), lipoprotein(a); oxL5, oxidized L5; oxLDL, oxidized low-density lipoprotein; oxLp(a), oxidized lipoprotein(a); oxPL, oxidized phospholipids; PAFR, platelet-activating factor receptor; PI3K, phosphatidylinositol-3-kinase; PLG, plasminogen; ROS, reactive oxygen species; SMC, smooth muscle cells; TNF-α, tumor necrosis factor alpha; tPA, tissue plasminogen activator; VCAM-1, vascular cell adhesion molecule-1

A large, growing body of experimental evidence suggests that both Lp(a) and L5 are important components in the pathogenesis of atherothrombotic disease (Fig. 2). The atherogenicity of Lp(a) may stem from its initiation of endothelial dysfunction, as shown in altered vascular reactivity in patients with elevated Lp(a) levels [51]. Like L5 and oxLDL, Lp(a) may accumulate in the arterial wall by binding to the subendothelial matrix elements, including fibronectin, fibrinogen, and proteoglycans, through lysine-binding domains found in apo(a). Although circulating plasma Lp(a) particles are less numerous than LDL particles, Lp(a) can become trapped in arterial walls through the binding of apo(a) to extracellular matrix proteins leading to high levels of Lp(a) within the wall [52••]. Notably, Lp(a) has been demonstrated to initiate alterations within the arterial wall that foster progression toward atherosclerosis [52••]. *In vitro* studies have shown that Lp(a) promotes the growth of ECs and smooth muscle cells [53]. Apo(a) contains several domains that are homologous to growth factors, but their functions are unknown [54]. Lp(a) is more readily oxidized than LDL, which may facilitate its uptake by macrophages via scavenger receptors [55]. Lp(a) also carries oxPL [56] and is covalently linked to apo(a). Having an endogenous danger-associated molecular pattern, oxPL can be recognized by cells of the innate immune system, triggering inflammation and, over time, calcification [56], which is important in the pathogenesis of ASCVD and calcific aortic valve disease.

Apo(a) is found in human aortic valves with atherosclerosis and calcification [10, 57], which involves mineralization and ossification of aortic valves. Patients with elevated levels of oxPL-apoB100 or Lp(a) showed enhanced uptake of ^18^F-NaF [58], indicating increased calcific activity, and had poorer clinical outcomes than those with normal levels [59].

Apart from its role in reducing fibrinolysis, Lp(a) may also induce platelet aggregation (Fig. 2). Apo(a), via kringle domains containing lysine-binding moieties, interacts with fibrinogen/fibrin and ECs to inhibit plasmin-mediated fibrinolysis *in vitro*. Although Lp(a) decreases fibrinolysis, its exact role in thrombus formation is unknown and still an area of ongoing research.

L5 has several atherogenic properties, including its proinflammatory characteristics associated with innate immune response activation (Fig. 2). L5 stimulates immune system components involved in the acute and chronic phases of atherosclerosis [60]. Moreover, L5 is prothrombotic and augments ADP-induced platelet activation and aggregation via LOX-1 and platelet-activating factor receptor (PAFR) [61]. An important step in atherosclerosis initiation and progression is the apoptosis of vascular ECs, and the proapoptotic effect of naturally occurring L5 is as potent as the artificially prepared oxLDL [4]. L5 promotes apoptosis by reducing the production of fibroblast growth factor-2 (FGF2) at the transcriptional level [4].

Because L5 can promote inflammation and increased cell permeability in vascular ECs, it may be responsible for initiating atherosclerosis [62]. Elevated L5 levels have been identified in some patient populations at high risk for CVD [63], suggesting that the increased electronegativity of LDL may be a reliable predictor of atherosclerosis. Because the methods currently used to measure circulating L5 require days to complete, rapid quantitative tests must be developed to perform large-scale epidemiologic studies to validate published observational results [62].

L5 may work with CRP [64] to induce atherosclerosis. In addition to its role as a marker of systemic inflammation, CRP may be a causative factor of vascular inflammation in atherosclerosis [65]. Although CRP is principally produced by hepatocytes after stimulation by inflammatory cytokines [66] (hence its role as a marker of inflammation), *in vitro* experiments showed that human aortic ECs also express CRP after stimulation with L5 [64]. This response depended on both the dosage and duration of L5 exposure, with CRP expression detected as early as 30 minutes after L5 exposure. Therefore, LOX-1, CRP, and L5 may collectively contribute to promoting atherogenesis.

## Lp(a) and L5 in Metabolic Syndrome and Diabetes Mellitus

The relationship between DM and Lp(a) is interesting and important. In a recent multicenter prospective analysis in patients who had chronic coronary syndrome, investigators found that patients who had impaired glucose regulation and elevated Lp(a) had higher event rates than did those with normal glucose regulation [67]. Lp(a) concentrations have been associated with type 1 DM complications. In a recent large study in patients with type 1 DM, the risk of complications was higher in patients with higher Lp(a) quartiles than in those with lower Lp(a) quartiles [68]. Cohort studies have shown increased cardiovascular events in prediabetic patients and patients with DM [67, 69]. In a meta-analysis, high Lp(a) levels were related to an increased risk of major coronary events and CVD events in patients who had type 2 DM compared with patients without DM [70]. Carotid atherosclerosis in patients with type 2 DM was significantly associated with increased Lp(a) levels, independent of conventional cardiometabolic risk factors. In addition, increased Lp(a) levels were related to carotid atherosclerosis, even when targeted concentrations of LDL-C were achieved [71•].

The genetic makeup of Lp(a) has been associated with the onset of DM and the regulation of circulating Lp(a) levels. Notably, the apo(a) kringle V- and IV-like domains exhibit structural similarity to plasminogen kringle V- and IV-like domains; each harbors a potential N-linked glycosylation site, suggesting a complex interplay in modulating physiological processes. Glycation can increase plasminogen activator inhibitor-1 formation while reducing tissue plasminogen activator synthesis induced by glycated lipoprotein (Lp(a)) in both venous and arterial ECs. This effect may be facilitated by EC-mediated oxidative modification and the generation of advanced glycation end products, which further promote the modification of tissue plasminogen activator and plasminogen activator inhibitor-1 production induced by glycated Lp(a) [72]. In addition, the mixture of hyperglycemia and high Lp(a) levels may reduce EC-derived fibrinolysis, which may in turn stimulate atherosclerotic changes and thrombosis in patients with DM [73].

L5 percentages of total LDL and/or L5 concentrations have been related to the severity of metabolic syndrome (MetS) in individuals with stable CVD [74]. The unique physicochemical and biologic properties of L5 in patients with DM suggest that L5 may increase the risk of atherosclerosis in this group. Yang et al. [15] showed that L5 molecules from patients with type 2 DM have proapoptotic properties, a higher protein concentration, and lower cholesteryl ester concentrations than L5 from healthy individuals. They also showed that all LDL subfractions from patients with DM induced apoptosis in vascular ECs to a greater extent than did LDL subfractions from healthy individuals. Furthermore, L5 from patients with DM was more effective in inducing bovine aortic EC apoptosis than were other fractions of LDL isolated from patients with DM or control individuals [15]. Corresponding deleterious outcomes *in vivo* may explain the cardiovascular damage observed in patients with DM.

From a mechanistic standpoint, treating ECs with pan-caspase inhibitor z-VAD-FMK immediately before exposure to L5 prevented L5-induced apoptosis, suggesting that the proapoptotic effect of L5 is caspase dependent. Lu et al. [75] showed that L5 derived from patients with DM induced EC apoptosis. This effect was attributed to the inhibition of FGF2 autoregulatory mechanisms, which would lead to disruption of collateral formation and FGF2-dependent reendothelialization. Independent of its oxidation state, L5 interrupts FGF2 autoregulation through the FGF2-PI3K-Akt loop mediated by the LOX-1 receptor, thereby inducing EC apoptosis and inhibiting endothelial progenitor cell differentiation (Fig. 2) [4, 75–77]. Ultimately, L5 in patients with MetS may increase markers of atherosclerosis while inhibiting the RXRα, RARα, CRBP1, and STRA6 cascades [78]. Alterations in the STRA6 cascade may be required in the formation of L5-induced atherosclerotic changes.

Studies have shown that plasma L5 percentages are comparable in patients with DM and those with hypercholesterolemia, regardless of LDL cholesterol levels [62]. Future research focused on determining the structural, molecular, and concentration-specific differences in L5 among healthy individuals and patients with DM or MetS will provide crucial insights.

## Lp(a) and L5 in Ischemic Stroke and Acute Myocardial Infarction

In recent years, studies have shown that high Lp(a) levels are related to stroke [79, 80] and calcified aortic valve stenosis [81]. In case-control and prospective studies, increased Lp(a) levels were independently and significantly associated with a high risk of ischemic stroke [82, 83] in both Asian and Caucasian populations [84•]. Elevated Lp(a) concentrations were also linked to higher risks of left atrial appendage thrombus formation, predisposing individuals to conditions such as atrial fibrillation and stroke, including intracerebral hemorrhage. In addition, Lp(a) concentrations were higher in Asian populations than in Caucasian populations, suggesting that Asian populations carry a greater risk of ischemic stroke [84••]. In meta-analysis studies, a relationship between high Lp(a) concentrations and ischemic stroke risk was identified after data were pooled from nested case-control, prospective-cohort, and case-control studies [85, 86]. Lp(a) is causally related to an increased risk of myocardial infarction, with a 2.3-fold increased likelihood of MACE reported when plasma Lp(a) levels exceed 30 mg/dl [87]. Data from genome-wide association and PROCARDIS (Precocious Coronary Artery Disease) cohorts linked 2 *LPA* single nucleotide polymorphisms to increased Lp(a) levels and coronary artery disease risk [88]. In observational studies, individuals with Lp(a) concentrations >50 mg/dL showed a 20% higher risk of ischemic stroke [79], with a hazard ratio of 1.34 compared to the low-Lp(a) group after adjustment [80]. Mendelian randomization analysis involving over 400,000 individuals revealed a positive correlation between Lp(a) levels and ischemic stroke due to aortic occlusion, and a negative correlation with stroke due to small vessel occlusion [89].

Genetic sampling from the Copenhagen City Heart Study and the Copenhagen General Population Study showed that elevated levels of Lp(a) and corresponding genetic variants of *LPA* were related to a high risk of ischemic stroke [79]. High Lp(a) levels have also been associated with ischemic stroke in young adults and children [90]. Consistent with these reports, increased Lp(a) was identified as an independent risk factor for ischemic stroke in a meta-analysis of prospective and observational studies, which may be particularly important in young patients with stroke [85].

Several groups have partially identified the underlying mechanisms of the association between acute ischemic stroke and L5. Shen et al. [61] showed that plasma L5 levels are increased in patients with acute ischemic stroke. In an experimental model of ischemic stroke, the treatment of wildtype mice with L5 resulted in larger infarct volumes than in control LOX-1^-/-^ mice. Furthermore, LOX-1 neutralization or deficiency after focal cerebral ischemia resulted in a 3-fold reduction in infarct volume, indicating a significant role of LOX-1 in stroke injury [61]. In the same study, investigators examined the synergistic effect of amyloid beta (Aβ) and L5 on the thrombotic pathway leading to stroke and found that L5, but not L1, induced the release of Aβ from platelets via signaling mechanisms involving IκB kinase 2 (IKK2). Furthermore, Aβ plus L5 synergistically stimulated the activation of GPIIb/IIIa receptors; the phosphorylation of c-Jun N-terminal kinase 1, p65, IκBα, and IKK2; and platelet aggregation [61]. The inhibition of NF-κB, LOX-1, or IKK2 prevented these outcomes. Furthermore, mice administered L5 plus Aβ had a 50% reduction in tail bleeding time; this effect was avoided by co-administering an IKK2 inhibitor [61].

The mechanism by which aspirin protects humans against ST-segment elevation myocardial infarction (STEMI) is unknown but may involve changes in gene expression or nitric oxide formation that are independent of its antiplatelet effects. Chang et al. [91] demonstrated that small concentrations of aspirin attenuated not only L5 incorporation into cells but also L5 cytotoxicity [64]. In another study, stroke patients exhibited a notable prevalence of electronegative LDL, regardless of high-dose statin therapy and other lipid-related factors [92].

Areas of critical research interest are the identification of plasma factors that can directly or indirectly trigger platelet activation and the development of novel targeted therapeutic approaches for STEMI. Chang et al. [91] found that patients with STEMI had higher circulating L5 concentrations than healthy individuals with minimal or undetectable levels; STEMI patients had a plasma L5% of approximately 15.4%, which was 10-fold higher than that in non-STEMI patients. Conceivably, L5 stimulates *FGF2* promoter methylation [75, 91], consequently reducing the production of FGF2, which is recognized as vital for EC function. In other work, L5 was isolated from the plasma LDL of patients with STEMI and injected into the tail vein of mice, which led to shortened tail bleeding time and platelet activation [93]. These findings indicate that L5 has prothrombotic effects. Furthermore, L5 from patients with STEMI increased the expression of P-selectin and tissue factor in ECs, promoted EC-platelet interactions, increased thrombocyte aggregation, and improved ADP-driven thrombocyte induction via LOX-1 and PAFR-mediated protein kinase Cα signaling pathways [92]. Through these complex interactions, L5 appears to contribute to the formation of blood clots that eventually lead to STEMI.

## Approaches to targeting Lp(a) and L5

Elevated levels of modified lipoproteins such as Lp(a) and L5 in patients with DM or MetS contribute to macro- and microvascular complications, increasing CVD risk. These lipoproteins may directly affect the vasculature or immune response. Genetic and epidemiologic studies suggest the need for therapies targeting modified lipids. Developing treatments to reduce Lp(a) and L5 is crucial for potentially reducing cardiovascular events [94•].

Circulating Lp(a) is often insensitive to fibrates or statins but can be reduced by high-dose niacin (2-3 grams/day) [95]. Although plasma apheresis can decrease Lp(a) levels by more than 50% [96], this expensive and time-consuming procedure is usually reserved only for patients with severe types of hypercholesterolemia, such as FH homozygosity. Trials in postmenopausal women undergoing estrogen replacement therapy showed considerable reductions in Lp(a), especially in women with higher baseline levels [97]. Because the *APOA* gene consists of an estrogen receptor response element, estrogen may act by reducing hepatic apo(a) secretion. Experiments conducted in HepG2 cell cultures showed that estrogen reduces expression of the apo(a) protein [98]. Estrogen therapy, however, is controversial, given the potential cardiovascular and cancer risks.

Because apo(a) is contained in Lp(a) and L5, attenuating apo(a) synthesis in the liver [99] may decrease the levels of both atherogenic entities; this could be achieved by using either antisense oligonucleotide or short-interfering RNA approaches [94•]. In a phase I trial, a GalNAc-conjugated antisense oligonucleotide (pelacarsen, known as IONISAPO(a)Rx) was administered subcutaneously at different doses and showed a strong reduction in Lp(a) of up to nearly 80% [100]. In a randomized, double-blind, placebo-controlled, dose-finding trial, siRNA against apo(a) (olpasiran, 10-mg doses for different time intervals) significantly decreased Lp(a) levels in patients with established ASCVD who had an Lp(a) concentration >150 nmol/L [101••].

Circulating Lp(a) levels correlate with apo(a) synthesis and are minimally affected by Lp(a) breakdown. Pharmacological activation of farnesoid X receptor (FXR) is a novel treatment avenue for high Lp(a) levels, potentially reducing coronary events in high-risk individuals [102]. FXR agonists have shown promise in reducing atherosclerosis in mice and reversing dyslipidemia in rodent models. FXR activation decreased plasma apo(a) concentrations, indicating potential Lp(a)-lowering effects [102]. Although traditional dyslipidemia drugs have a limited impact on Lp(a), emerging therapies such as PCSK9 inhibitors, cholesteryl ester transfer protein inhibitors, and second-generation antisense oligonucleotides show promise in lowering elevated Lp(a) levels [103•].

Determining the distinct features of L5 in patients with DM, hypertension, or stroke, and in healthy individuals, may open new avenues for developing therapeutic approaches targeting L5. Currently, there are no clinical trials aimed at lowering L5 levels; however, ongoing preclinical research is actively exploring the mechanisms of L5 formation and how levels are regulated. Although blocking LOX-1 can interrupt L5’s signaling, this approach may lead to systemic side effects since LOX-1 also has a high affinity for other electronegative substances, heparin, bacteria, and CRP due to the LOX-1 lectin like domain [104]. Accordingly, the removal of L5 from the plasma or directly targeting L5 in the circulation may be the most efficient strategy for preventing atherosclerosis. Recently, we found that treatment with atorvastatin for 6 months (10 mg/day) partially reduced plasma L5 concentrations in patients with hypercholesterolemia, with a rebound of L5 after therapy noncompliance [64]. Although statins contribute to the partial reduction of L5 levels, further strategies are required to effectively decrease L5 levels.

## Conclusions

The development of ASCVD is closely linked to DM and MetS and involves disturbances in lipoprotein metabolism, notably in Lp(a) and L5, which contribute to pathophysiology. Lp(a) stands out as a genetically determined risk factor for CVD; strong epidemiological evidence supports its association with calcific aortic valve disease and ASCVD. Its proinflammatory and calcific properties link it to early atherosclerotic disease. Genetic studies further confirm its role in cardiovascular risk. Clinical trials suggest additional benefits from reducing Lp(a) alongside LDL-C. Despite its clinical significance, Lp(a) is often not measured before or after ASCVD events. L5, a modifiable lipoprotein associated with atherosclerosis progression, deserves attention in this area. Despite decades of research, quantifying L5 remains challenging, limiting its evaluation in clinical trials. Improving methods of detection and further understanding L5's role in ASCVD may lead to the development of novel therapeutic agents targeting its pathways.

## Data Availability

Not applicable.

## Abbreviations

Aβ: Amyloid Beta
apo(a): Apolipoprotein (a)
apoAI: Apolipoprotein AI
apoB100: Apolipoprotein B100
apoCIII: Apolipoprotein CIII
apoE: Apolipoprotein E
ASCVD: Atherosclerotic Cardiovascular Disease
CRP: C-Reactive Protein
DM: Diabetes Mellitus
ECs: Endothelial Cells
FGF2: Fibroblast Growth Factor 2
FH: Familial Hypercholesterolemia
FXR: Farnesoid X receptor
HDL: High-Density Lipoprotein
ICAM-1: Intercellular Adhesion Molecule 1
IKK2: IκB Kinase 2
IL: Interleukin
L1: The Least Electronegative LDL (Regular Low-Density Lipoprotein)
L5: The Most Electronegative LDL
LDL: Low-Density Lipoprotein
LDLR: Low-Density Lipoprotein Receptor
LOX-1: Lectin-Like Oxidized Low-Density Lipoprotein Receptor-1
Lp(a): Lipoprotein(a)
Lp-PLA2: Lipoprotein-Related Phospholipase A2
lysoPC: Lysophosphatidyl Choline
MetS: Metabolic Syndrome
NEFA: Nonesterified Fatty Acids
oxL5: Oxidized L5
oxLDL: Oxidized Low-Density Lipoprotein
oxLP(a): Oxidized Lipoprotein(a)
oxPL: Oxidized Phospholipids
PAFR: Platelet-Activating Factor Receptor
PAF-AH: Platelet Activating Factor-Acetyl Hydrolase
pI: Isoelectric Point
PI3K: Phosphatidylinositol-3-Kinase
ROS: Reactive Oxygen Species
sdLDL: small-dense Low-Density Lipoprotein
STEMI: ST-Segment Elevation Myocardial Infarction
TG: Triacylglycerol
TNF-α: Tumor Necrosis Factor-Alpha
VLDL: Very Low-Density Lipoprotein
